# A Quantitative Study of the Relationship between the Distribution of Different Types of Collagen and the Mechanical Behavior of Rabbit Medial Collateral Ligaments

**DOI:** 10.1371/journal.pone.0103363

**Published:** 2014-07-25

**Authors:** Chao Wan, Zhixiu Hao, Shizhu Wen, Huijie Leng

**Affiliations:** 1 State Key Laboratory of Tribology, Tsinghua University, Beijing, China; 2 Department of orthopedics, Peking University Third Hospital, Beijing, China; University of Minho, Portugal

## Abstract

The mechanical properties of ligaments are key contributors to the stability and function of musculoskeletal joints. Ligaments are generally composed of ground substance, collagen (mainly type I and III collagen), and minimal elastin fibers. However, no consensus has been reached about whether the distribution of different types of collagen correlates with the mechanical behaviors of ligaments. The main objective of this study was to determine whether the collagen type distribution is correlated with the mechanical properties of ligaments. Using axial tensile tests and picrosirius red staining-polarization observations, the mechanical behaviors and the ratios of the various types of collagen were investigated for twenty-four rabbit medial collateral ligaments from twenty-four rabbits of different ages, respectively. One-way analysis of variance was used in the comparison of the Young's modulus in the linear region of the stress-strain curves and the ratios of type I and III collagen for the specimens (the mid-substance specimens of the ligaments) with different ages. A multiple linear regression was performed using the collagen contents (the ratios of type I and III collagen) and the Young's modulus of the specimens. During the maturation of the ligaments, the type I collagen content increased, and the type III collagen content decreased. A significant and strong correlation (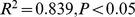) was identified by multiple linear regression between the collagen contents (i.e., the ratios of type I and type III collagen) and the mechanical properties of the specimens. The collagen content of ligaments might provide a new perspective for evaluating the linear modulus of global stress-strain curves for ligaments and open a new door for studying the mechanical behaviors and functions of connective tissues.

## Introduction

Ligaments play a vital role in maintaining the stability and normal function of musculoskeletal joints. During recent decades, numerous studies have focused on the function, injury and healing of ligaments from different perspectives, such as clinical treatments, biochemical responses and the mechanical properties of tissues. The mechanical properties of ligaments have attracted much interest given their key contributions to the structural responses of ligaments as well as normal joint function [1]–[3]. Numerous factors have been examined to determine their effects on the mechanical properties of ligaments; these factors include water content [4], fibril crimp [5], [6], fiber orientation [7], [8], fibril diameter and density [9], [10], age [11], [12], sex [13], [14] and body and ligament anthropometry [15].

Ligaments are biological composite materials that generally consist of ground substance, collagen and elastin fibers. Very few elastin fibers exist in skeletal ligaments (less than 1% of the weight of the solid intercellular substance) and approximately 80% of the intercellular solid substance is composed of collagen fibers [16], [17]. Types I and III collagen are the major constituents of collagen fibers, with type I collagen accounting for approximately 90% and type III accounting for the remainder [16]. The distributions of type I and type III collagen were measured in rabbit medial collateral ligaments (MCLs) in our previous study, which revealed that the type I collagen content was significantly lower in the core portion of the ligaments than at the periphery (P<0.005) [18]. The type III collagen molecule is more flexible than the type I collagen molecule; thus, fibers rich in type III collagen are more extensible than those rich in type I collagen (i.e., type III collagen is less stiff than type I collagen) [17], [19]. Therefore, the inhomogeneous distributions of the various types of collagen can partially account for the heterogeneous material properties of ligaments.

However, whether the levels of the various types of collagen are related to the material properties of ligaments remains unclear. Both the tensile stretch and failure force of aponeurotic scar tissue correlate with the type I and III collagen contents [20], but these two properties (i.e., the stretch and failure force) are not representative of material properties because of their dependence on tissue geometry. In other papers, collagen types were suggested to be qualitatively related to the mechanical properties of ligaments, and the material stiffness of healed ligaments was reduced, possibly because type III collagen synthesis increases during the early phases of ligament healing [21]–[25]. By contrast, Ng et al. [26] reported that a significant negative correlation between the type III collagen content and the Young's modulus of ligaments did not exist. Quantitative studies of the relation between the different collagen types and the material properties of ligaments will help to better answer this question.

The site of ligament rupture, the value of surface strain and the mechanical properties of ligaments all change with age (i.e., during the maturation of ligaments). Woo et al. [11], [12] investigated the tensile properties of MCLs from rabbits of different age groups and reported a significant increase in the tensile modulus of ligaments as a result of maturation and subsequent aging. Lam et al. [27] found that both the strain value at failure and the rupture location changed greatly with rabbit maturation and suggested that the changes would be due to the heterogeneous material properties of ligaments. Fibrillogenesis and mRNA levels of collagen contents changed during the maturation of the rabbit MCL and patellar tendon [28]. Kostrominova and Brooks [29] also found that the amount of type III collagen increased in older tendons compared with young tendons. It has also been demonstrated that ligaments become weaker, as measured by their mechanical properties, while they are healing because type III collagen is more rapidly generated than other types. During long-term healing, the rapidly generated type III collagen is replaced by type I collagen, and the material properties of ligaments can recover to their normal levels [21]–[24]. However, whether changes in the mechanical properties of ligaments during aging (i.e., maturing) are the result of the variations in the different collagen contents must be clarified.

In this paper, the content of different types of collagen and the mechanical properties of ligaments were determined for the MCLs taken of rabbits of various ages. We hypothesized that the ratios of type I and III collagen would change with ligament growth and maturation and found that the collagen contents were correlated with the material properties of the ligaments. The content of different types of collagen could serve as a new perspective to evaluate the mechanical behaviors of ligaments.

## Materials and Methods

### Tissue retrieval and specimen preparation

All procedures were approved by the Experimental Animal Care and Use Committee of Tsinghua University. Twenty-four healthy male New Zealand white rabbits from the experimental animal center of Tsinghua University were euthanized with a lethal dose of carbon dioxide, and the animals were separated into a 1.5-month-old group (n = 6, weight 1.33±0.25 kg), a 4- to 5-month-old group (n = 6, weight 2.71±0.32 kg), a 6- to 7-month-old group (n = 6, weight 3.09±0.27 kg), and a 12- to 13-month-old group (n = 6, weight 3.82±0.53 kg). A previous paper reported that the changes in body anthropometry for rabbits tapered off after the age of 6 months [11]; thus, the first three ages corresponded to three phases during the maturation of rabbits, and the 12- to 13-month-old group corresponded to mature rabbits. One intact hind leg was harvested from each animal randomly and was double-wrapped in gauze (wet with phosphate buffered solution (PBS)). All the whole limbs were sealed in airtight plastic bags at −20°C until testing.

Before testing, the intact hind limbs were thawed at room temperature for 24 h. The MCL with attached bones (i.e., femur and tibia) were resected from the knee joints of the hind legs. It was known that a thin delicate translucent membrane (called ‘epiligament’) covered the surface of ligament. The epiligament tissue was more mobile and compliant than the underlying ligament, but was weakly and intimately adherent to the surface of the ligament [30]. This induced the strain from the calculation of random speckles on the epiligament tissue to be not representative of the actual performance of the ligament tissue. Therefore, the epiligament tissues (sheath) of MCL were striped carefully by surgeons using scalpel for measuring the mechanical properties of ligament tissue properly.

**Figure 1:**
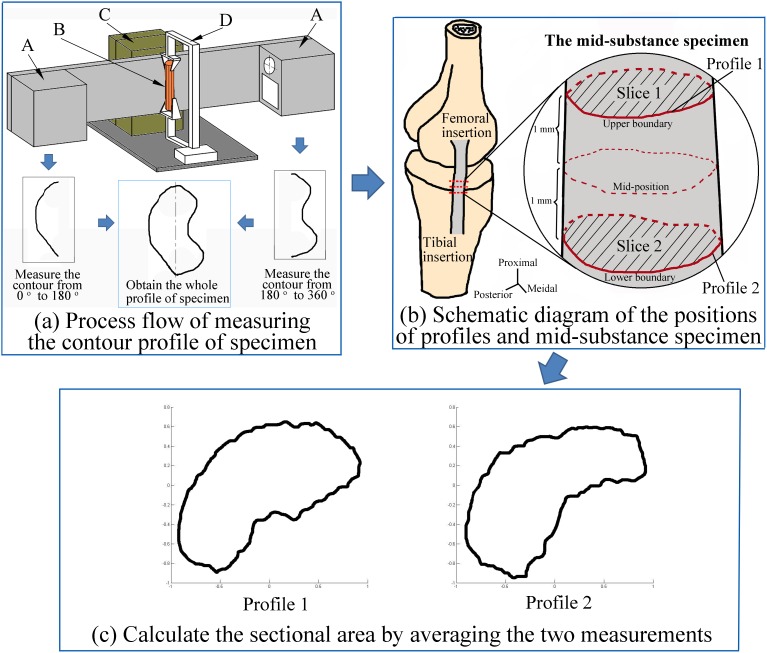
The methodology and devices for measuring the sectional areas of specimens. (a) Bone-ligament-bone samples (B) were fixed by clamps (D) with their longitudinal axes parallel to the direction of the motion unit (C). The contour profiles of the mid-substance specimen between 0–180 degrees and 180–360 degrees were measured by the two opposite sensors (A), respectively. And the whole profile could be obtained by combining the two separate profiles. (b) With the two sensors moving along the direction of the motion unit, two whole profile measurements (i.e., profile 1 & 2) were acquired at the cross sections that were 1 mm far from the mid-position. The mid-position was located at the central section between the femoral and tibial insertions. (c) An average sectional area for each mid-substance specimen was calculated by the two whole contour profiles.

The specimens were defined as the mid-substance between two sections which were 1 mm far from the mid-position of MCL (shown in Figure 1(b)). All the mechanical properties of the mid-substance specimens were first determined from the whole bone-MCL-bone samples using a mechanical testing machine, and the mid-substance specimens were then resected from the whole ligaments and sectioned to observe the distribution of the different types of collagen. To determine the mechanical properties and the collagen content for the same specimen, all the mechanical tests were performed within the range of elastic deformation to maintain the tissue integrity of the ligament sections for further observation.

### Biomechanical analysis

A custom device system was built to measure the contour profiles of the specimens (Figure 1(a)). The system consisted of a 1-dimension motion unit (GZ130DY30J-F, Beijing Opgher Instrument Co., Ltd, China), two 2-dimension laser profile sensors (ZG2-WDS22, OMRON IAB Group, Japan), sensor controllers (ZG2-WDC-11, OMRON IAB Group, Japan), an optical table, and a laptop computer. Given the limited measuring range of the laser profile sensor (180 degrees), the two 2-dimension laser profile sensors were mounted opposite each other to allow for measurement of the complete cross-sectional profile of the specimens. The resolutions of the sensor were 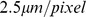 and 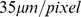 across the height and width of the sensor, respectively. The resolution of the 1-dimension motion unit was 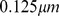. With the sensors mounted in the motion unit, two complete contour profiles were obtained at the two sites that were 1 mm far from the mid-position of each ligament (‘profiles 1 & 2’ in Figure 1(b)). Using a custom-written MATLAB script (The MathWorks Corporation, USA), two cross-sectional areas were calculated from the two complete contour profiles (Figure 1(c)), and an average area was determined from these two areas for characterizing the dimension of each mid-substance specimen. This custom-built measuring system was validated before it was used to obtain the contour profiles of the specimens. In the validation experiments, one plastic cylinder (approximately 6 mm in diameter) was measured by a microcaliper and this custom-built device nine times for each method. The average value of the nine measurements was used for each measuring method. The difference between the results of the two different measuring methods was only 0.2% and was not significant (P>0.05), demonstrating the accuracy of this measuring methodology in our study. The nine area results from our custom device system were 27.54±0.1211 mm^2^, and the low standard deviation (approximately 0.44% of the mean value) demonstrated the repeatability of this method.

**Figure 2:**
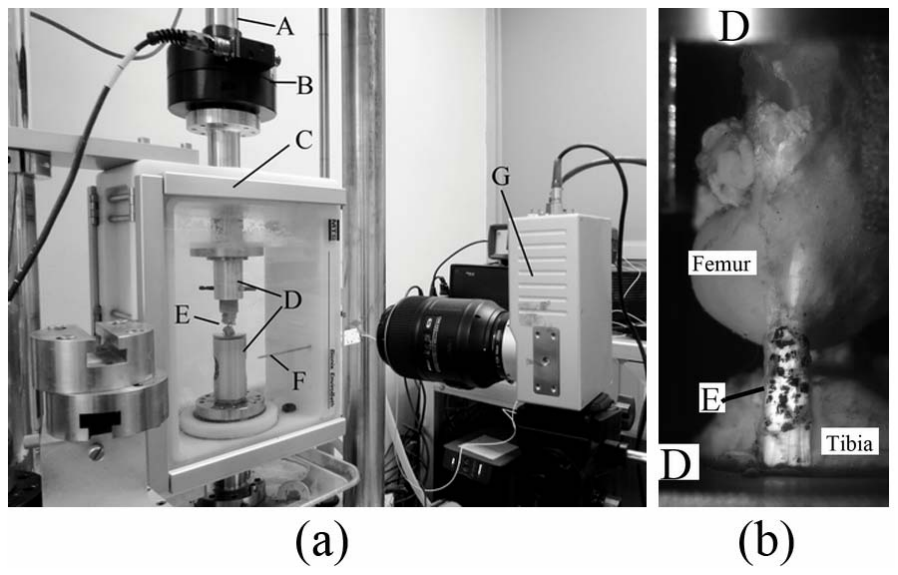
Experimental apparatus used to measure the mechanical behavior of the mid-substance specimen of rabbit MCL. The tensile load was applied by an actuator (A), and recorded by a load cell (B) in the MTS testing machine. Two clamps (D) were adjusted to hold the bone-MCL-bone sample (E) vertically to ensure that the tensile load was applied along the longitudinal axis of the mid-substance specimen of the ligament. All the tests were performed in a bath chamber (C) at physiological temperature (controlled by a temperature sensor (F)) and humidity. The displacements of ink speckles on the mid-substance specimen were measured by a camera (G) and used to calculate the tensile strain by a custom-written MATLAB script.

After the measurement of the sectional area, the femur and tibia bones of the bone-MCL-bone samples were embedded in polymethylmethacrylate and rigidly fixed by custom-designed clamps on a material testing machine (MTS, Eden Prairie, MN, USA). Adjustments to both the embedment and the clamp fixation subjected the mid-substance specimens to an axial tensile deformation with their fibers vertically oriented. All tests were performed inside a bath chamber with a constant temperature of 37°C and PBS bathing (Figure 2(a)). Random speckles were marked on the surface of the mid-substance using black oil ink (Figure 2(b)). Images (matrix 1280×1024) of the speckle displacements were recorded using a digital camera (CR600×2, Optronis GmbH, Kehl, Germany). The testing protocol was similar to that reported in the previous paper [9]. A small preload of 1 N was applied. Then, cyclic preconditioning (between 0 mm and 0.5 mm extension) was performed at an extension rate of 10 mm/min for 10 cycles. After the preconditioning, the bone-MCL-bone samples were extended at a rate of 0.1 mm/s, and simultaneously, the displacement of the speckles on the mid-substance was recorded by the camera. The load data and the speckle images were collected at 50 Hz by the MTS material testing machine and the camera, respectively. Additionally, the longitudinal orientations of each mid-substance specimen in the initial and the final CCD images were compared to determine whether the mid-substance specimens subjected to an axial tensile deformation. The angle results between the two orientations of each specimen were 1.26±1.07 degree (Mean±SD), which demonstrated that the specimens were almost applied by an axial tensile load.

An open-source MATLAB program for digital image correlation and tracking [31] was used to calculate the strain results of the mid-substance specimens using the speckle images. To validate the program, five bone-shaped plastic samples (length = 180 mm, width×thickness = 13 mm×5 mm) were tested under an axial tensile load, and the region of interest of each sample was covered with random speckles. An average strain was calculated by the MATLAB program for the region of interest on each sample. An extensometer (MTS, Eden Prairie, MN, USA) was also mounted on the sample to measure the strain in the region. A linear regression between the strains measured using the two methods revealed that the strain values calculated by the MATLAB program were significantly and highly correlated with those measured by the extensometer (*Strain-by-random-speckles*  = −0.0009+0.974*Strain-by-extensometer, R^2^ = 0.9922, P<0.0001). All the validation tests of the plastic samples were performed according to the ASTM standard (ASTM D638-10).

**Figure 3:**
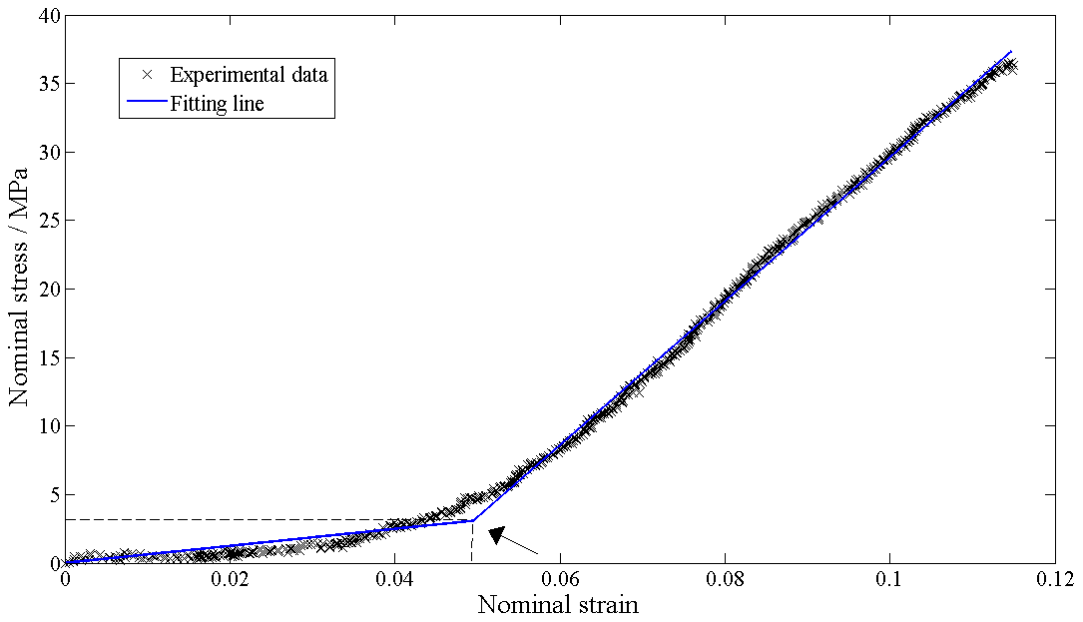
Example bilinear curvefitting of stress-strain tensile experimental data. The arrow indicates the transition point of the fitting line from the toe-region to the linear region.

Using the validated program, an average nominal strain could be determined for each mid-substance specimen. The nominal stress values of the mid-substance specimens were calculated by dividing the load values by the initial average cross-sectional area. In accordance with the assumption of bilinear curve fitting for the stress-strain tensile data of ligaments [32], [33], the stress-strain curves of the specimens were fitted by bilinear lines (Figure 3). The Young's modulus in the linear region of the stress-strain curve of each mid-substance specimen (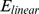) was quantified to represent the mechanical behavior of the mid-substance of MCL.

### Measurements of collagen contents

The picrosirius red staining-polarization method can distinguish and determine the level of types I and III collagen and has been used for many tissues, such as skin [34], [35], lung [36], cartilage [37], periodontal ligament [38], vocal ligament [39], and tendon [19], [40]. As described in our previous study [18], the type I and III collagen contents in the mid-substance of the ligament were determined by this method. First, all the mid-substance specimens were resected from the bone-MCL-bone samples, fixed in Bouin's fluid for 24 h, dehydrated with graded ethanol solutions, and embedded in paraffin. Two paraffin slices (5 µm slice thickness) were extracted from each specimen by sectioning the paraffin blocks using a microtome (Leica RM 2235, Leica Corporation, Germany). The two paraffin slices corresponded to the upper and lower boundaries of the mid-substance specimens in the biomechanical analysis (Figure 1(b)). The paraffin slices were stained with 0.1% picrosirius red solutions (Sirius Red F3B, Sigma, USA) and observed under a polarized light microscope (Leica DMI6000B, Leica, Germany). The preparation of the tissue specimens and the settings in the staining procedure were both the same as those in previous papers [41], [42].

**Figure 4:**
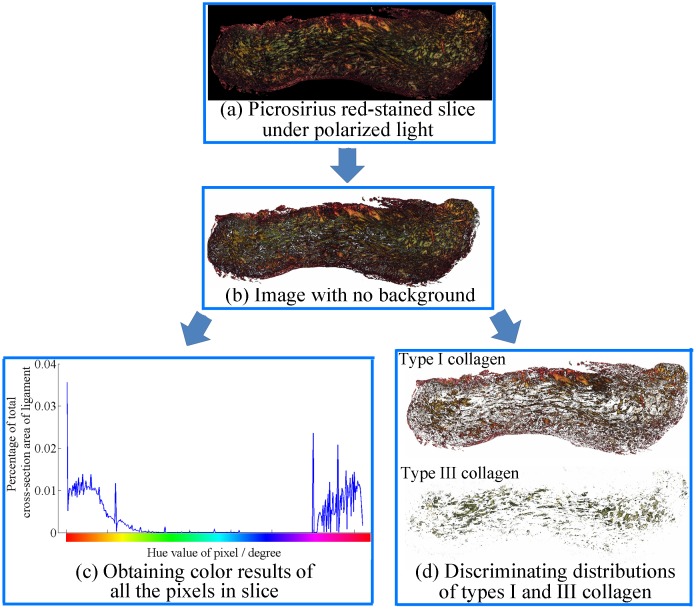
A process flow diagram for differentiating the collagen contents in the histological slice of the mid-substance specimen. Two slices were sectioned at the upper and lower boundaries of each mid-substance specimen, corresponding to the locations that 1 mm far from the mid-position of the ligament, respectively (shown as ‘slice 1 & 2’ in Figure 1). Each slice was firstly stained with 0.1% picrosirius red solutions, and imaged under polarized light (a). Then, the dark background of the image was removed for further image processing (b). The color results of all the pixels in each slice were obtained (c): the type I and type III collagen corresponded to the pixels with red-yellow color (hue values of 345–75 degrees) and greenish color (hue values of 75–165 degrees), respectively. Average collagen ratios were calculated from the collagen content values in the two slices for characterizing each mid-substance specimen. In addition, the distributions of types I and III collagen in the specimen slice were determined by the hue values of the pixels (d).

Because of the difference in the birefringence of various collagen types, type I and type III collagen appeared as red-yellow and green colors, respectively, under a polarized light microscope [41], [42]. The type I/III collagen could then be distinguished by the hue value of each pixel in the images using hue-saturation-brightness (HSB) color model. The hue variable was expressed as a chromatic circle (0–360 degrees). The red-yellow color and the greenish color were defined as the hue values between 345–75 degrees and 75–165 degrees, respectively. The process flow of calculating the different types of collagen in each slice is presented in Figure 4.

For the pixel-based method used for distinguishing colors, the effect of the magnification factor (i.e., the resolution of one pixel) on the distribution of the two types of collagen should be eliminated. Four images of the same sample were photographed using different magnification factors (×25, ×100, ×200, and ×630). The pixel matrix of all the images was 2040×1536. The resolutions were 2506 nm/pixel, 626 nm/pixel, 313 nm/pixel, and 99 nm/pixel for the ×25, ×100, ×200, and ×630 magnifications, respectively. A difference of less than 0.5% was found in the proportions of the different collagen types between the images under the ×200 and ×630 magnifications. Thus, the ×200 magnification was validated to be sufficiently accurate for discriminating collagen types of specimens.

With the same algorithm developed in our previous study [18], the cross-sectional area of the whole tissue, 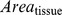, the area of the type I collagen, 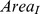 (i.e., yellow-red pixels), and the area of the type III collagen, 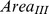 (i.e., greenish pixels), were calculated for each slice automatically. The ratios of type I and III collagen were defined as 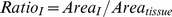 and 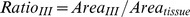, respectively. The 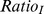 and 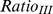 values for each mid-substance specimen were determined by averaging the corresponding values of the two slices of each specimen.

### Statistical analysis

One-way analysis of variance (one-way ANOVA) was used to identify any significant differences in the 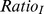, 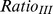, and 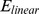 values among the four different age groups (i.e., the 1.5-, 4- to 5-, 6- to 7-, and 12- to 13-month-old groups). A Bonferroni adjustment was chosen for all the multiple comparisons. A multiple linear regression was performed between 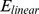 and the two factors (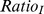 and 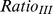) to determine the relationship among these three variables. The variance inflation factor (VIF) was also calculated to estimate the multicollinearity of the two factors. Significance for all the tests was defined as P<0.05.

## Results

**Figure 5:**
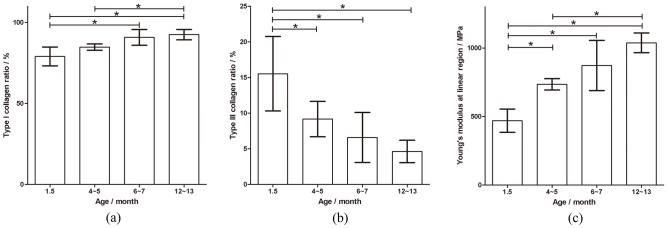
Comparisons of the ratio of type I collagen (a), the ratio of type III collagen (b), and the Young's modulus in the linear region of the stress-strain curve (c) between the different age groups. During the maturation of ligaments (i.e., from the 1.5-month-old group to the 12- to 13-month-old group), the ratio of type I collagen increased while the ratio of type III collagen decreased. Significant differences were found in the ratio of type I collagen between the 1.5-month-old group and the 6- to 7-month-old group, between the 1.5-month-old group and the 12- to 13-month-old group, and between the 4- to 5-month-old group and the 12- to 13-month-old group (P<0.05). The ratio of type III collagen in the 1.5-month-old group was significantly higher than that in the other groups (P<0.05). The Young's modulus in the linear region changed significantly between the 4- to 5-month-old group and the 12- to 13-month-old group as well as between the 1.5-month-old group and the other three groups (P<0.05). Mean±SD.

The age and body mass of the rabbits, and the cross-sectional area,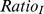, 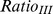, and 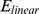 of the mid-substance specimens are summarized in Table 1. The multiple comparison results of the one-way ANOVA for the 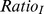, 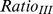, and 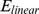 values among the different age groups are presented in Figure 5. Both the type I and III collagen contents of the specimens changed with the ligament growth and maturation. The ratios of type I collagen (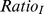) and type III collagen (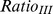) were 79.0±5.82% and 15.5±5.22% for the 1.5-month-old group, 84.8±1.92% and 9.17±2.47% for the 4- to 5-month-old group, 90.8±4.83% and 6.59±3.51% for the 6- to 7-month-old group, and 92.5±3.19% and 4.62±1.58% for the 12- to 13-month-old group, respectively (presented as mean±SD). Significant differences for 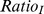 were observed between the 1.5-month-old group and the 6- to 7-month-old group, between the 1.5-month-old group and the 12- to 13-month-old group, and between the 4- to 5-month-old group and the 12- to 13-month-old group (P<0.05). Compared with the 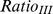 of the 1.5-month-old group, the 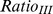 of the other groups was significantly reduced (P<0.05). Moreover, no significant differences were found in the 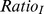 and 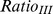 values between the 4- to 5-month-old group and the 6- to 7-month-old group as well as the 6- to 7-month-old group and the 12- to 13-month-old group.

**Table 1:** The age and body mass of rabbit, and the cross sectional area, collagen contents (ratio of type I collagen, 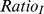, and ratio of type III collagen, 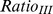) and mechanical property (Young's modulus in the linear region of the stress-strain curve 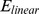) of the mid-substance specimens of MCL.

No.	Age/Month	Body mass/Kg	Cross-sectional area/mm^2^	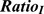 /%	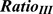 /%	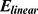 /MPa
M-1	1.5	1.48	1.23	70.0%	24.4%	318.5
M-2	1.5	1.02	0.852	81.6%	15.7%	499.2
M-3	1.5	1.01	0.425	81.7%	15.0%	525.4
M-4	1.5	1.57	0.984	73.9%	9.04%	421.2
M-5	1.5	1.42	0.795	81.1%	17.2%	507.8
M-6	1.5	1.48	0.841	85.7%	11.9%	525.4
M-7	4	2.31	1.56	85.2%	11.6%	751.6
M-8	4	2.42	1.74	87.3%	7.99%	769.5
M-9	4	2.62	1.80	81.9%	4.98%	758.7
M-10	5	3.10	3.06	86.1%	9.36%	745.4
M-11	5	3.03	2.06	83.3%	11.6%	730.2
M-12	5	2.77	1.85	85.0%	9.58%	654.5
M-13	6	3.31	2.99	90.1%	6.59%	945.5
M-14	6	3.27	2.12	96.2%	3.01%	1069
M-15	6	3.14	1.53	84.8%	10.9%	874.9
M-16	6	3.31	1.57	96.6%	1.91%	1015
M-17	7	2.75	1.55	86.6%	9.30%	572.2
M-18	7	2.75	1.38	90.6%	7.84%	755.8
M-19	12	3.13	2.06	88.4%	5.70%	962.4
M-20	12	3.75	2.61	92.3%	5.18%	1007
M-21	12	4.59	4.42	92.0%	5.54%	999.6
M-22	12	3.67	2.52	95.3%	4.50%	1064
M-23	13	3.50	2.71	97.0%	1.52%	1169
M-24	13	4.25	2.46	90.0%	5.30%	1023

For the four different age groups, the Young's modulus in the linear region 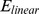 was 469.5±85.23 MPa, 735.0±41.57 MPa, 872.1±183.4 MPa, and 1037±72.43 MPa in order of increasing age (shown as mean±SD). 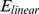 also increased significantly with age among the four groups (P<0.05) except between the 4- to 5-month-old group and the 6- to 7-month-old group and between the 6- to 7-month-old group and the 12- to 13-month-old group.

A multiple linear regression was performed between 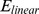 and the type I and type III collagen ratios. The VIF value of the two factors (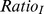 and 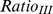) was calculated to be 3.255, indicating no multicollinearity between the two factors. The correlation equation was 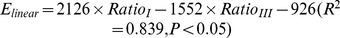. Both the high correlation coefficient (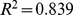) and the P-value (P<0.05) suggested a significant strong relationship between the mechanical behavior (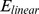) and the collagen contents of the ligaments (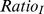 and 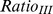). These findings indicated that 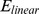 is reduced as 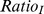 decreases and 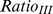 increases.

## Discussion

Mechanical properties are critical contributors to the structural response and function of ligaments, and numerous factors have been studied to determine their influence on the mechanical properties of ligaments [4]–[15]. The various types of collagen (including type I and III collagen) are the major constituents in ligaments, but whether collagen types are related to the mechanical behaviors of ligaments must still be clarified. To our knowledge, there are few quantitative studies that determine whether the collagen types are correlated with the material properties of ligaments. If a correlation exists, the mechanical behavior of ligaments could be evaluated on the basis of the different types of collagen contents, and the heterogeneous distribution of material properties in ligaments could be explained from a new perspective.

Types I and III collagen were distinguished by the picrosirius red staining-polarization method. It has been suggested that type I collagen is mainly thick and densely packed in thick fibrils, whereas type III collagen is mainly thin fibers, composed of loosely packed thin fibrils [41], [42]. Due to the differences in both the diameters and the aggregation patterns, type I and type III collagen appeared as a yellow-red color and a greenish color, respectively, using this method [18], [43]. The picrosirius red staining-polarization method results agreed with the immunohistochemical results for type I collagen [34] and type III collagen [44]. Hence, this method has been used to determine the collagen content in various soft tissues [19], [34]–[40].

The results indicated that the ratios of type I and type III collagen varied among the different age groups, as shown in Figure 5 (a) and (b), respectively. As the age increased from 1.5 months to 12–13 months, the ratio of type I collagen increased. By contrast, the type III collagen ratio decreased. The changes in type I and type III collagen during the maturation of healthy ligaments are similar to the variation in the collagen contents during the early phase of ligament healing [21]–[24]. The ratios for type I and type III collagen (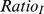 and 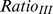) changed significantly among all the age groups except for the 4- to 5- versus 6- to 7-month groups and the 6- to 7- versus 12- to 13-month groups; this finding is potentially attributed to the decreased ligament growth observed with aging. Although the type I collagen continued to increase and the type III collagen continued to decrease with maturation, the decreased growth results in non-significant (P>0.05) changes between some age groups. Similarly, no significant changes occurred in the mechanical properties of the mid-substance of the ligaments between the 6- to 7-month-old group and the 12- to 13-month-old group (P>0.05) [11]. The ratios of the collagen contents in the 6- to 7-month-old group were consistent with previous measurements [45]. Normal ratios of type I and III collagen in the MCL of male New Zealand white rabbits (6- to 8-month-old) were obtained using a methodology that identified specific peptides [45]. The normal ratios of type I and III collagen, which correspond to the percentage of type I and III collagen in the total amount of these two collagen types, were 91±2% and 9±2%, respectively (mean±SE). For a comparison with their results, the normal ratios of type I and III collagen were calculated (93.2±1.51% and 6.80±1.51%, respectively) and no significant difference was found (P<0.05).

Significant correlation was observed between the collagen contents (including type I and type III collagen) and the mechanical properties of the ligaments (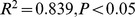). The regression equation indicates that the ligaments become stiffer as the ratio of type III collagen (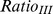) decreases. This change is not consistent with the observation that no significant correlation exists between the type III collagen and the mechanical behavior of ligaments [26]. This disagreement may be due to the difference in the methods of calculating the modulus in the two studies. The stress-strain curves of the ligaments included two regions, a toe region for small strains and a linear region after a transition point (shown in Figure 3). It was suggested that the toe region results from either the sequential recruitment of fibrils under tensile loads or the wavy geometry of collagen fibers [2], [46]–[48]. In the linear region, all the fibrils were stretched past the toe region; thus, the Young's modulus of the linear region was exclusively dependent on the material behavior of the fibrils in the ligaments. In the study by Ng and coworkers [26], a secant modulus was used, which provided a global indicator of tissue stiffness and included the effects of other factors, such as fibril crimp or fiber recruitment. The Young's modulus of the linear region in the stress-strain curve was used in our paper to eliminate the effect of fiber crimp or fibril recruitment. For ligaments composed primarily of type I collagen, 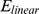 was calculated from the regression equation and was 1.2 GPa (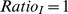). This value is consistent with the Young's modulus of type I collagen reported in another paper [49]. In that study, the type I collagen was extracted from sea cucumber and tested using a novel microelectomechanical systems (MEMS) technique. It was reported that the Young's modulus was 0.86±0.45 GPa under a low strain of approximately 0.09. Reese and coauthors also assumed a similar value for the collagen fiber (i.e., 1 GPa) when simulating the micromechanical behavior of the helical superstructures in ligaments [46]. Briefly, the high correlation value suggests that the ratio of collagen contents could be considered a novel perspective for evaluating the mechanical behaviors of ligaments. The ligaments became stiffer as the ratio of type I collagen increased or the ratio of type III collagen decreased.

The Young's modulus in the linear region of the stress-strain curve increased from youth to maturity (Figure 5(c)). The Young's modulus of the 1.5-month-old group was significantly reduced compared with the other groups (P<0.05). The modulus of the 4- to 5-month-old group was also significantly reduced compared with the 12- to 13-month-old group (P<0.05). Similar results reported that the Young's moduli of MCLs were 520 MPa, 674 MPa, 800 MPa, and 940 MPa for 1.5-month-old, 4- to 5-month-old, 6- to 7-month-old, and 12- to 13-month-old groups, respectively [11]. The moduli results for the corresponding groups in our paper were 469.5±85.23, 735.0±41.57, 872.1±183.4, and 1037±72.43 MPa, these values indicate slightly more stiffness than their findings. Rat tail tendons also become stiffer during postnatal development, and this finding was suggested to result from a qualitative increase in the collagen fibril diameter [50], [51]. Nevertheless, whether the fibril diameter is related to the material properties of ligaments has still not been agreed upon. Some researchers suggested no significant correlation between fibril diameter and the Young's modulus of ligaments [52], [53], whereas a significant correlation was reported in other papers [54], [55]. Because of both the significant strong correlation between the collagen types and the mechanical properties of ligaments and the weak correlation between collagen types and fibril diameters [18], the change in the mechanical behavior of the maturing ligaments may be more adequately explained by variations in the collagen types.

However, some limitations are found in this study. First, the experimental specimens were from a single source (i.e., all samples were harvested from healthy individuals), although rabbits with different ages were included. The lack of healed/injured specimens may limit the applicability of the conclusions to healthy ligaments. Some healed/injured samples will be included in the future to quantitatively study the relation for abnormal ligament tissues. Second, the strengths of the cross-links in different samples were assumed to be identical, and the influence of different cross-linking strengths was neglected in this paper. Although cross-links have been suggested to be critical to the mechanical properties of collagenous tissues (f.i., patellar tendon) [56], the stiffness of collagenous tissues is determined by the material behavior of individual collagen fibrils rather than other factors, such as interfibrillar cross-links and/or interfibrillar glycosaminoglycan bridges [57].

In summary, the results for the mechanical behaviors and the collagen contents were obtained from rabbit MCLs of different ages using an axial tensile test and a picrosirius red staining-polarization method, respectively. The Young's modulus in the linear region of the stress-strain curves and the ratios of type I and III collagen for the different age groups were analyzed using one-way ANOVA and multiple linear regression. The type I collagen content increased with ligament maturation, whereas the ratio of type III collagen decreased. In the study of the relationship between the collagen contents and the mechanical behaviors of ligaments, the ratios of type I and type III collagen (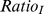 and 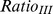) were determined to be significantly and strongly related to the Young's modulus in the linear region of the stress-strain curve 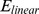 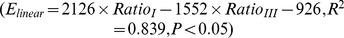. Despite some limitations of this study, these findings potentially open a new door for predicting and evaluating the mechanical behaviors of ligaments in the future.
